# iPSCs in NK Cell Manufacturing and NKEV Development

**DOI:** 10.3389/fimmu.2022.890894

**Published:** 2022-07-08

**Authors:** Nicholas Boyd-Gibbins, Peter Karagiannis, Do Won Hwang, Shin-Il Kim

**Affiliations:** ^1^ THERABEST Japan, Inc., Kobe, Japan; ^2^ Center for iPS Cell Research and Application (CiRA), Kyoto University, Kyoto, Japan; ^3^ Research and Development Center, THERABEST Co., Ltd., Seoul, South Korea

**Keywords:** natural killer cells, extracellular vesicles, exosomes, induced pluripotent stem cells, manufacturing, genome engineering, immunotherapy, cancer

## Abstract

Natural killer (NK) cell immunotherapies for cancer can complement existing T cell therapies while benefiting from advancements already made in the immunotherapy field. For NK cell manufacturing, induced pluripotent stem cells (iPSCs) offer advantages including eliminating donor variation and providing an ideal platform for genome engineering. At the same time, extracellular vesicles (EVs) have become a major research interest, and purified NK cell extracellular vesicles (NKEVs) have been shown to reproduce the key functions of their parent NK cells. NKEVs have the potential to be developed into a standalone therapeutic with reduced complexity and immunogenicity compared to cell therapies. This review explores the role iPSC technology can play in both NK cell manufacturing and NKEV development.

## Introduction

Natural killer (NK) cell adoptive cell transfer (ACT) is emerging as an important cancer immunotherapy. Despite engineered T cell therapies advancing through clinical trials to commercialization (1), some major challenges remain such as high rates of serious adverse side effects, production inefficiencies, and high costs for autologous treatment generation (2). Recent research has shown that NK cells can overcome these challenges to develop into an independent or complementary class of cancer immunotherapies (3–6). A complementary field benefiting from advances in NK cell development is that of NK cell extracellular vesicles (NKEVs), with purified NKEVs having proven to reproduce key functions of their parent NK cells (7).

These developments coincide with induced pluripotent stem cell (iPSC)-derived cell therapies reaching human clinical trials. A particular focus is on iPSC-derived cell products that can be given to patients allogeneically, reducing long-term risks that have slowed translation. iPSC-derived allogeneic cell therapies have the potential to create “off-the-shelf” products, allowing larger batches to be created, reducing costs, and increasing reproducibility. Together, these developments set the scene for iPSC technologies to offer advantages in the manufacture and translation of NK cell-based therapeutics.

## NK Cells

NK cells are members of the innate lymphoid family, identified as CD56^+^CD3^-^, which provide frontline defense against infections and cancer, and clear damaged cells (8, 9). NK cells are typically classified into two main subpopulations: Cytotoxic CD56^dim^CD16^+^ (CD56^dim^) NK cells account for ~90% of the total NK population, and IFN-γ-producing immunoregulatory CD56^bright^CD16^-^ (CD56^bright^) NK cells make up the remaining ~10%(10).

NK cells can discriminate normal from abnormal cells, a process called immune surveillance, *via* a repertoire of activating and inhibitory receptors (11–13). Direct binding to target cell ligands by a combination of natural cytotoxicity receptor (NCR) family members and NKG2D stimulate NK cell activation and the trafficking of constitutively expressed lytic granules to the site of cell contact and into the target cell (14–16) or the secretion of cytokines (5, 17). Alternatively, CD16 binding alone is sufficient to activate antibody-dependent cellular cytotoxicity (ADCC) (11).

To protect host cells, HLA class I molecules are selectively detected by NK cell inhibitory receptors such as NKG2A and killer immunoglobulin-like receptors (KIRs) (18). Other NK cell inhibitory receptors detect sialic acid, extracellular matrix components, and aminophospholipids, and the expression of immune checkpoints by NK cells, such as CTLA-4 and PD-1, can be stimulated by specific signaling environments (18, 19).

NK cells can be used allogeneically for ACT due to their ability to educate and establish “self-tolerance” to the host HLA class I environment (20). The major sources for NK cell ACT have been peripheral blood (PB-NK) and cord blood (CB-NK), as well as the immortalized line NK-92 (21, 22). Both PB-NK and CB-NK cells are derived from limited donor sources, introducing batch-to-batch variation. NK-92 cells possess anti-cancer potential (23, 24), and have shown efficacy in human clinical trials (25). However, they lack CD16 expression and require irradiation prior to transplantation to inactivate proliferation (26), which in turn impairs therapeutic properties (27, 28). iPSC-derived NK (iPSC-NK) cells have the potential to overcome these limitations while offering additional advantages (29).

## Enhancing NK Cell Function

**Figure 1:**
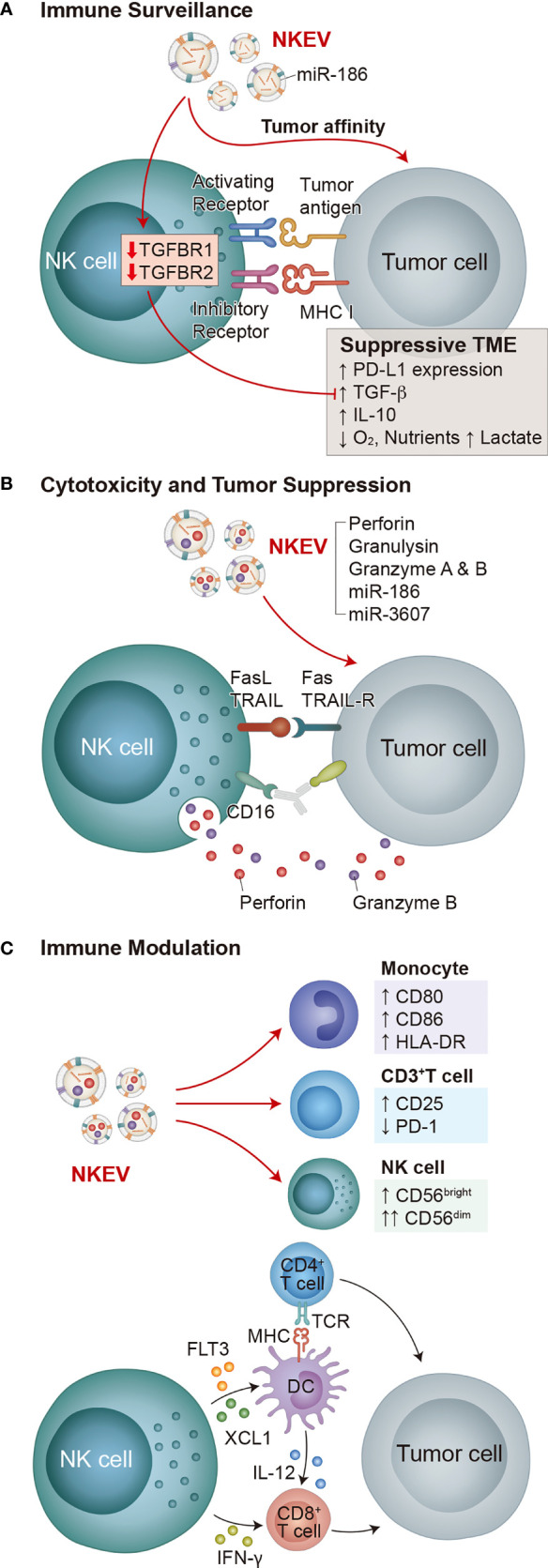
Functions of NK cells and NKEVs. The key functions of NK cells are immune surveillance, cytotoxicity and tumor suppression, and immune modulation, all of which involve both NK cells and NKEVs. **(A)** NK cell immune surveillance depends on the interaction of activating and inhibitory receptors with target cells. In suppressive TMEs, NK cells are inhibited by PD-L1 on tumor cells and tumor cell-secreted TGF-β and IL-10. Decreased oxygen concentration, increased lactate production, and decreased available nutrients also inhibit NK cells. NKEVs exhibit tumor affinity, and NKEV delivery of miR-186 to NK cells decreases TGFBR1/2 expression, fortifying them in suppressive TMEs. **(B)** Cytotoxicity and tumor suppression by NK cells depends on either CD16 regulated ADCC or degranulation of vesicles containing perforin and granzyme B in response to the combined activation of activating receptors. NKEVs directly deliver cytotoxic effector cargo of perforin, granulysin, granzyme A and B, as well as miRNAs miR-186 and miR-3607 to tumor cells. **(C)** NK cells produce immunomodulatory cytokines and chemokines in response to activation, directly activating CD8^+^ T cells, and stimulating dendritic cells to activate both CD8^+^ and CD4^+^ T cells, which subsequently attack tumors. Meanwhile, NKEVs increase CD80, CD86 and HLA-DR expression on monocytes, increase CD25 expression and decrease PD-1 expression on CD3^+^ T cells, and increase the total NK cell population and the CD56^dim^ NK cell fraction.

Over time, tumors develop immunosuppressive microenvironment features (30) (**Figure 1A**) such as altered expressions of receptors and ligands that activate or inhibit NK cells (18, 31, 32), the recruitment of immunomodulatory cells into the tumor mass (33), altered metabolism that results in lower oxygen and increased lactate (34, 35), and the production of inhibitory molecules including TGF-β, IL-10, PGE2, and immune checkpoint proteins such as PD-L1 (32, 36).

Considering these immune evasion mechanisms, various strategies to enhance NK cell function have been developed (4, 37–39). Research into the optimal selection and dosing regimes of cytokines used for expansion and activation (39–42) has identified IL-15 as the preferred choice (43), while work on membrane bound IL-15 and IL-21 has also shown advantages (44–46). Cancers with decreased IL-15 expression correlate with decreased patient survival (47), which led to the development of IL-15 superagonists (48–50) and NK cell modifications that can overcome TGF-β-mediated inhibition of the IL-15 pathway (51, 52).

Another approach has been co-treatment with small molecule immunomodulatory drugs that activate NK cells and increase granzyme-B expression (53, 54) or that increase the expression of NK activating ligands on cancer cells (55, 56). Increased NK cell effects have also been achieved with the combined use of biologics, such as tumor-specific monoclonal antibodies (mAbs) that augment NK cell functions (57–63), or *via* bi-specific or tri-specific engagers that bind to tumor-specific antigens and NK cells to form immunological synapses (64–67).

Immune checkpoint blockade (ICB) deploying mAbs to block inhibitory pathways has revolutionized our approach to cancer treatment. PD-1/PD-L1 blockade has been shown to increase NK cell cytotoxicity against cancer cells (68–70), and combined treatment of lung cancer patients with allogeneic PB-NK cells and the ICB drug Pembrolizumab increased patient survival (71). Other ICB targets have been identified, and multiple mAbs targeting inhibitory NK cell pathways have reached human clinical trials (72).

Finally, the growing importance of genetic strategies to enhance NK cell function (73), as discussed later, has brought iPSCs to the forefront of NK cell production (29).

## Extracellular Vesicles

Extracellular vesicles (EVs), including endosome-derived exosomes (40-150 nm) and plasma membrane-derived microvesicles (50-1000 nm), are lipid nanoparticles secreted by most cell types that are involved in intercellular communication (74). Due to the difficulty determining the biogenesis pathway of individual vesicles, they are classified according to size or density, biochemical composition, or descriptions of conditions of the cell of origin (e.g. "NKEVs") (75).

In recent years EVs have become a major area of research interest. For mesenchymal stem cells (MSCs), it became clear that their immunomodulatory and regenerative functions primarily act through secretory paracrine pathways, including *via* EVs (76, 77). EVs have the potential to reproduce features of many parent cell therapies while potentially simplifying translational pipelines due to low immunogenicity and inability to replicate (78, 79). Furthermore, progress has been made in EV manufacturing and storage (80–84), critical areas for EV translation.

Mostly using MSC-EVs (85), interventional human clinical trials are active for dystrophic epidermolysis bullosa (NCT04173650), regeneration of macular holes (NCT03437759), acute ischemic stroke (NCT03384433), periodontitis (NCT04270006), craniofacial neuralgia (NCT04202783), inflammatory lung diseases (NCT04388982, NCT04276987), and neurodegenerative diseases (NCT04202770, NCT04388982). T-cell-derived EVs are being investigated for pneumonia (NCT04389385). EVs are also being investigated as liquid biopsy markers for diseases such as cancer (NCT04053855, NCT04523389, NCT04852653, NCT04529915, NCT03228277), diabetes (NCT03106246), neurodegeneration (NCT03944603), and panic disorder (NCT04029740).

## Natural Killer Cell Extracellular Vesicles

Although NKEVs have yet to reach clinical trials (85), they have become a significant research focus (7). NKEVs are continuously produced by NK cells and are involved in key mechanisms of NK cell function including immune surveillance, cytotoxicity, and immune modulation (86–90) (**Figure 1**).

NKEVs express NK markers such as CD56, NKG2D, and cytotoxic effector proteins (e.g. perforin, granzymes A and B, granulysin, and FasL) (86, 87), as well as EV markers Rab5B, CD63, CD81, CD9, and TSG101 (87). Purified NKEVs are cytotoxic against diverse cancer cells (**Figure 1B**) including hematological cancers (Jurkat, K562, DAUDI) (87), neuroblastoma (CHLA-136) (91), breast carcinoma (MCF-7 (91), MDA-MB-231/F) (92)), ovarian cancer (A2780) (93), and melanoma (B16F10) (94). In mouse glioblastoma xenograft models NKEVs exhibit tumor affinity (92, 95) (**Figure 1A**).

As well as NK cell effector proteins, NKEVs carry miRNAs that have specific roles in cancer suppression. For example, Sun et al. showed that miR-3607, enriched in purified NKEVs, was required for NK cells to inhibit the malignant transformation of pancreatic cancer cells (Mia PaCa-2, PANC-1) by directly targeting IL-26, suppressing proliferation, migration, and invasion (96). Neviani et al. showed that miR-186 in NKEVs is partially responsible for their cytotoxic effect against neuroblastoma cells (CHLA-136, CHLA-255, and LAN-5) while fortifying other NK cells against the suppressive effect of TGF-β (97) (**Figure 1A**). NKEVs containing miR-207 have also been shown to reduce neuroinflammation (98).

NKEVs contain immunomodulatory proteins (88, 99, 100) and promote M1 macrophages in a mouse pseudomonas aeruginosa-induced lung injury model (101), reproducing immunomodulatory features of NK cells. Federici et al. reported that NKEVs stimulate CD25 expression on CD3^+^ T cells, HLA-DR and costimulatory molecule expression on monocytes, and increase the total NK cell population and the CD56^dim^ NK cell fraction *in vitro (* 88) (**Figure 1C**). Shoae-Hassani et al. showed that NK cells cocultured with neuroblastoma cells (SK-N-SH and CHLA-255) produce NKEVs that confer enhanced neuroblastoma cell cytotoxicity to fresh NK cells (102).

EVs may lack the signaling or metabolic pathways required to respond to inhibitory tumor microenvironment (TME) signals. Accordingly, some groups have shown experimentally that NKEVs retain tumor affinity, tumor suppressive, and immunomodulatory properties in simulated immunosuppressive TMEs using TGF-β, IL-10, and LPS (88, 97). The addition of NKEVs also reduced PD-1 expression on CD3^+^ T cells even in the presence of TGF-β and IL-10 (88).

Overall, mounting evidence suggests that NKEVs are an integral component of NK cell functions (39, 103), with purified NKEVs demonstrating therapeutic properties (7, 39).

## Priming NK Cells for EV Production

NKEVs collected from IL-15-primed NK cells had increased concentration of cytotoxic effectors (92), improved cytolytic activity against cancer cells of glioblastoma, breast cancer, and thyroid cancer, showed improved tumor affinity, and inhibited glioblastoma growth in xenograft mice (92). In NK cells IL-15 regulates the small GTPase Rab27a (92), which was shown in MSCs to increase EV secretion by promoting maturation of endosomal multivesicular bodies (MVBs) containing exosomes (104). Zhu et al. showed similar effects in NK cells with IL-15 priming more than doubling particle number and EV-contained protein (92).

Hypoxic TMEs suppress NK immune surveillance *via* hypoxia-induced tumor cell shedding of MICA and MICB (105, 106), inhibit NK-mediated cell killing by reducing KIR expression (106), decrease intracellular perforin and granzyme B concentration (107), and reduce degranulation (106). Yet CD16 function is largely maintained, facilitating ADCC (106). To compound this, the NK cell response to the hypoxic TME actually assists blood vessel maturation (108). However, activity of the hypoxia-induced HIF-1α pathway promotes the infiltration of NK cells into tumors and the expression of granzyme B (108). Away from the TME, NK cells cultured in hypoxia for 48 hours produce larger yields of NKEVs with increased total protein, FasL, perforin, and granzyme B concentrations, increased cytotoxicity against breast (MCF-7) and ovarian (A2780) cancer cells *in vitro*, and increased inhibition of the migration and proliferation of these cancer cells (93). These results are similar to the effects of IL-15 priming, and the two approaches have been shown to be synergistic (109).

Harnessing these NK priming approaches and developing knowledge in this area, as well as more generally into the conditions that maximize NKEV yield and potency, may prove critical in NKEV manufacturing optimization, as seen for other EV sources.

## iPSCs in NK Cell Manufacturing

iPSC-derived cell therapies are now featured in many clinical trials, including those using iPSC-NK cells (NCT04106167, NCT03841110) (110). The expansion potential of iPSCs eliminates the need for multiple donors, increasing cell product reproducibility, and epigenetic rejuvenation during iPSC reprogramming erases DNA modifications, producing cells that are biologically young (111–113). This has been shown to cause immune cells to exit exhausted states and adopt phenotypes effective at killing cancer cells (114).

For NK cell-based ACT, chemically defined differentiation protocols have been used to produce iPSC-NK cells with cytotoxicity and immunomodulatory function comparable to primary NK cells (29, 115–117). In cancer patients NK cells are known to undergo functional decline (118–120), similar to that observed in aged patients (121). This decline suggests iPSC-NK cell therapies have advantages, where biologically young, functional cells can replenish the diminished NK cell activity of older and sicker patients.

One of the key advantages of using iPSC technology for cell therapy is its suitability to genome engineering (122). A myriad of genetic NK cell enhancement strategies have been developed to improve targeting and homing to cancer cells, resist immunosuppressive TMEs, and increase cytotoxicity and persistence (73).

For example, deletion in iPSCs of *CISH*, which encodes the CIS protein, a negative regulator of IL-15, resulted in iPSC-NK cells with better metabolic fitness and increased IL-15 sensitivity (123). Another example is the addition to iPSCs of a cleavage resistant CD16 variant that resulted in enhanced iPSC-NK cells with superior ADCC compared to both unmodified iPSC-NK cells and primary NK cells, and caused comparatively more regression of hematopoietic malignancies and solid tumors when combined with a mAb treatment (124).

Chimeric antigen receptor (CAR)-NK cells, emerging as a key area of cancer immunotherapy development, are also better suited to using iPSC technology. Despite clinical approval of autologous CAR products, allogeneic products avoid patient cell morbidity due to aging or disease and the possible contamination of cancer cells (125). Using iPSC technology has allowed researchers to compare the effectiveness of CAR combinations (116), and two CAR iPSC-NK cell clinical trials are underway (NCT04245722 and jRCT2033200431).

In addition to genome engineering, iPSCs are compatible with synthetic biology. Tumor-derived TGF-β suppression of NK cell cytotoxicity (47) is ameliorated by knocking out TGF-β receptors (51). Intracellularly, TGF-β upregulates miR-27a-5p (126), which if inhibited also increases the cytotoxicity of NK cells (127). Intracellular targets like miR-27a-5p can be targeted by miRNA switches to enhance NK cell function in a context-dependent way. miRNA switches are synthetic mRNAs that can activate the expression of specific miRNAs or proteins in response to endogenous biomolecules (128). Moreover, miRNA switches can be designed to orthogonally, meaning multiple miRNA switches can be combined to tune NK cell cytotoxic and metabolic (129) responses to specific signaling environments. Employed in iPSC-NK cells, this approach could be used to engineer “intelligent” NK cells with programmed context-dependent functions.

## iPSCs in NKEV Development

**Figure 2:**
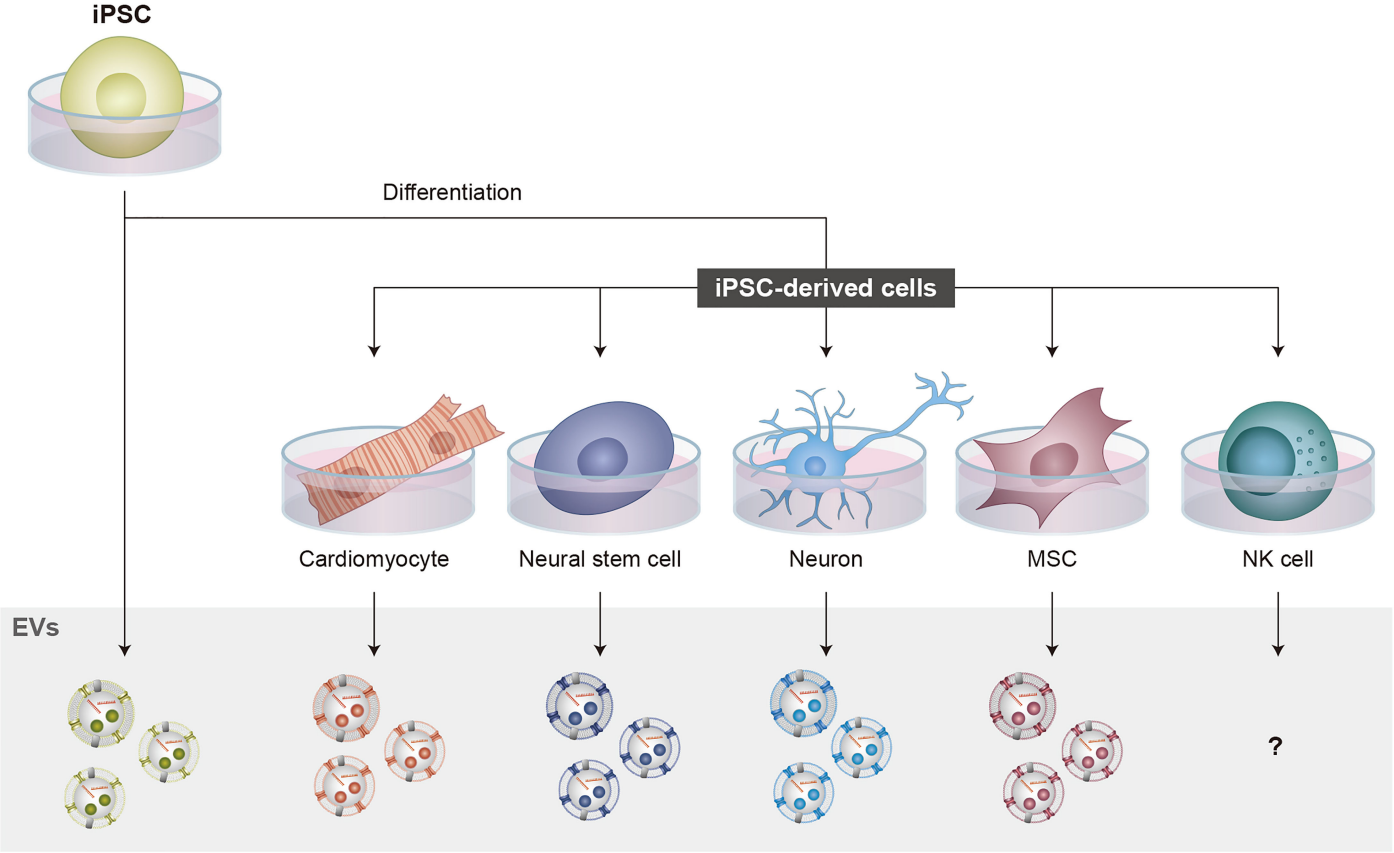
iPSCs in EV production. iPSCs can be differentiated into various cell types that have therapeutic potential. iPSC-NK cells have the advantages of increased expansion potential, the production of biologically young cells, and less donor variation compared to primary NK cells. For EV production, several iPSC-derived cells and iPSCs themselves have been shown to produce functional EVs. However, for NK cells, studies investigating iPSC-NKEVs have not been reported, raising the important question of whether iPSC-NK cells produce EVs. NKEVs can reproduce the functions of NK cell therapies while reducing the complexity and immunogenicity of the final therapeutic product, thus increasing safety. These features highlight how iPSC-NKEVs represent an important direction for NKEV research.

While research on NKEVs has increased, investigations into iPSC-NK cell-derived EVs (iPSC-NKEVs) remain unreported, raising the question of whether iPSC-NK cells also produce EVs (**Figure 2**). This may represent an important, currently underexplored therapeutic opportunity considering recent research from both NKEVs and EVs from other iPSC-derived cells (130, 131).

Therapeutic properties of iPSC-derived cardiomyocyte- (132–134), neuron- (135), neural stem cell- (136, 137), and MSC- (138–141) EVs, as well as iPSC-EVs (142–144), have already been demonstrated, as has the potential to improve performance for certain applications using bioengineering (145). Using iPSCs as a source for EV production may also help address EV manufacturing and translational challenges such as heterogeneity and scalability (146).

For iPSC-NK cells, Cichocki et al. have shown that they can reproduce key features of NK cells, including dose-dependent cytotoxicity against diverse cancer cells (lung carcinoma (A549), hepatocyte carcinoma (HepG2), ovarian adenocarcinoma (SKOV-3), myeloid leukemia (K562), and melanoma (SK-MEL2)), inflammatory cytokine production, *in vivo* immunomodulation (including activation and recruitment of circulating T cells), infiltration into solid tumor spheroids *in vitro*, and the ability to slow tumor progression *in vivo* (115). Other groups have reported functional iPSC-NK cells (116), and given the documented role of NKEVs in these NK cell processes, these results underline the importance of investigating iPSC-NK cells for EV production.

Similarly to the epigenetic rejuvenation discussed for iPSC-NK cells, Man et al. reported that epigenetic rejuvenation of osteoblast progenitors *via* histone deacetylase (HDAC) inhibition results in the production of EVs with enhanced function (147). Other studies have shown that, compared to older MSCs, young MSCs produce EVs with better therapeutic properties (148, 149) that are enriched in miRNAs and proteins involved in immunomodulation (148, 150, 151). Interestingly, studies directly comparing therapeutic potential have shown improved efficacy of iPSC-derived MSC-EVs compared to adult donor MSC-EVs in *in vitro* studies of wound healing (152) and in *in vivo* disease model studies of osteoarthritis (153). Together, these findings suggest that rejuvenated iPSC-derived cells may be a superior resource for EV manufacturing compared to other sources, although donor age prior to iPSC reprogramming does impact some EV properties (154).

Engineering EVs to increase potency and specificity has already shown promising results in other cell types, and the same principles may translate to NKEVs. Upregulated expression of miRNAs can increase the concentration of miRNAs in EVs, improving therapeutic performance (155–157). Clinical trials are in progress using modified EVs for drug delivery in pancreatic (NCT03608631), colon (NCT01294072), and lung cancer (NCT01159288) (85). While there are yet to be published reports of groups modulating the biochemical composition of NKEVs genetically, the principle of NKEV engineering has been established by Han et al., who used electroporation to load NKEVs with the chemotherapy drug paclitaxel, enhancing their ability to suppress the proliferation and induce the apoptosis of breast cancer cells (158). In MSCs, Böker et al. showed that overexpression of the EV tetraspanin CD9 resulted in increased exosome biogenesis (159), highlighting the role iPSC engineering can play in optimizing production efficiency as well as modulating EV composition.

## Conclusion and Future Directions

While the EV industry has moved into a phase of production optimization and human clinical translation, NKEVs are at an earlier stage of development. On the one hand, this means that they can benefit from advancements in purification, storage, and scale-up technologies, but, on the other hand, key translational questions remain relatively unanswered. One question concerns the extent of NKEV heterogeneity, and how this relates to NK cell sub-populations and states. Another concerns the production efficiency of NKEVs, which depends on their potency and yield, and ultimately the number of particles required for effective therapeutic doses. For EVs from other cell types, appropriate doses have been determined (160), and production yields have been documented and linked to manufacturing processes (80, 161). For NK cells, Jong et al. reported that 1-3 x 10^9^ activated NK cells cultured in the G-Rex100 culture system for 48 hours contained ~7 x 10^7^ particles/ml, implying production yields of ~5-14 particles/cell/day (91). Other cell types have been reported to have much higher EV production yields (161). Indeed, Jong et al. compared their NKEV production data to HEK293 (~1841 particles/cell/day) and MSCs (~938 particles/cell/day) (91). Further reports on NKEV production efficiency and detailed investigation of effective therapeutic doses will provide important context to these early numbers. If it is confirmed that iPSC-NK cells secrete NKEVs, then the ability to expand iPSCs to vast numbers before differentiation could be exploited for iPSC-NKEV production.

For NK cell ACT, iPSCs offer advantages in key areas of manufacturing and translation, promising to provide a cell source for biologically young, “off-the-shelf”, and bioengineered enhanced iPSC-NK cells. With iPSCs already making an impact in the clinic, iPSC-NK cells can benefit from advances in manufacturing (162) and genome engineering strategies (163) to create iPSC-NK cells that have context-dependent functions and enhanced potency and specificity. For NKEVs, future work may soon confirm that their composition can be genetically controlled, and, similarly to enhanced NK cells, this could lead to the development of enhanced NKEVs with the potential to be purified as a stand-alone therapeutic or deployed as an addition to engineered iPSC-NK cells that can home to tumor sites and secrete enhanced NKEVs *in situ*.

## Author Contributions

NBG, PK and SIK conceptualized the overall paper and NBG drafted the manuscript. NBG primarily researched and structured the EV sections, PK the iPSC-NK cell section, and SIK and DWH the NK cell biology and iPSC-NK sections. NBG and SIK conceptualized the figures. All authors contributed to reviewing and editing, and PK edited the English for the final submission. All authors contributed to the paper and approved the submitted version.

## Funding

This work was supported by a 2020 Gibon Yeongu Program from the National Research Foundation of Korea (NRF) funded by the Ministry of Education, Science, and Technology(2020R1F1A106727812).

## Conflict of Interest

NBG is the CSO and a board member of THERABEST Japan, Inc. SIK is the CSO and a board member of THERABEST Co., Ltd and the co-CEO and a board member of THERABEST Japan, Inc. DWH is the CTO and a board member of THERABEST Co., Ltd.

PK declares that the research was conducted in the absence of any commercial or financial relationships that could be construed as a potential conflict of interest.

## Publisher’s Note

All claims expressed in this article are solely those of the authors and do not necessarily represent those of their affiliated organizations, or those of the publisher, the editors and the reviewers. Any product that may be evaluated in this article, or claim that may be made by its manufacturer, is not guaranteed or endorsed by the publisher.
